# Efficacy and safety of vitamin D supplementation in diabetic kidney disease: an umbrella review of systematic reviews and meta-analyses

**DOI:** 10.3389/fneph.2026.1883351

**Published:** 2026-07-15

**Authors:** Víctor Juan Vera-Ponce, Jhosmer Ballena-Caicedo

**Affiliations:** Facultad de Medicina (FAMED), Universidad Nacional Toribio Rodríguez de Mendoza de Amazonas (UNTRM), Chachapoyas, Amazonas, Peru

**Keywords:** albuminuria, diabetic kidney disease, diabetic nephropathy, grade, proteinuria, systematic review, umbrella review, vitamin D

## Abstract

**Background:**

Diabetic kidney disease (DKD), including classical diabetic nephropathy, is a leading cause of chronic kidney disease and kidney replacement therapy. Vitamin D supplementation has been proposed to reduce albuminuria, but the available reviews are heterogeneous and redundant.

**Objective:**

To critically evaluate and summarize the efficacy and safety of native vitamin D and active vitamin D analogs in adults with DKD, including studies reported as diabetic nephropathy (DN).

**Methods:**

An overview of reviews registered in PROSPERO (CRD420251250914). Systematic reviews, with or without meta-analysis, were searched in MEDLINE/PubMed, Embase, the Cochrane Library, Web of Science, and Scopus, without language restrictions, through April 2, 2026. Methodological quality was assessed with AMSTAR-2, risk of bias with ROBIS, overlap with Corrected Covered Area (CCA), and certainty of evidence with GRADE. The synthesis used one anchor review per outcome, without *de novo* quantitative reanalysis.

**Results:**

Eleven reviews met the eligibility criteria; nine provided evidence derived from randomized trials or separable randomized-trial data, and two were retained as contextual evidence. GRADE certainty was low for the surrogate urinary outcomes UACR and UAER, low to very low for 24-hour proteinuria, and very low or not evaluable for renal function, kidney replacement therapy, mortality, cardiovascular events, safety, and metabolic-inflammatory outcomes. Overlap was high (overall CCA, 13.5%; RCT-derived corpus, 14.1%). The evidence was compatible with low-certainty reductions in surrogate urinary outcomes, especially UACR and UAER, without consistent benefit for eGFR, serum creatinine, kidney replacement therapy, mortality, or cardiovascular events; safety was insufficiently reported.

**Conclusions:**

Vitamin D shows a low-certainty reduction in surrogate urinary markers of albuminuria/proteinuria in DKD, but current evidence is insufficient to support vitamin D as a specific renoprotective intervention beyond conventional indications for deficiency or mineral and bone disorders.

**Systematic review registration:**

https://www.crd.york.ac.uk/PROSPERO/view/CRD420251250914, identifier CRD420251250914.

## 1 Introduction

Diabetes mellitus imposes a growing epidemiologic burden. In 2021, the Global Burden of Disease (GBD) study estimated 529 million people living with diabetes (95% uncertainty interval [95% UI]: 500–564 million), with a global age-standardized prevalence of 6.1% (95% UI: 5.8–6.5) and a projection exceeding 1.31 billion people by 2050 (95% UI: 1.22–1.39 billion) (1). The International Diabetes Federation reported 589 million adults aged 20–79 years living with diabetes in 2024 and projected 853 million by 2050 (2). In South and Central America, the corresponding figure was 35.4 million in 2024, with an expected increase to 51.5 million by 2050; in Peru, adult prevalence was 6.4%, equivalent to 1,335,800 affected adults (3, 4). The renal burden attributable to diabetes represents a substantial component of this morbidity and mortality: a GBD 2021 analysis of chronic kidney disease due to type 2 diabetes estimated 107.6 million prevalent cases and 11.28 million disability-adjusted life-years (DALYs) in 2021, with sustained increases in mortality and DALYs since 1990; notably, Andean Latin America showed the greatest percentage change in age-standardized mortality rate across regions, placing Peru in an epidemiologic setting of direct clinical relevance to this question (5).

Vitamin D has been the subject of clinical investigation in diabetic kidney disease because of its involvement in regulation of the renin–angiotensin–aldosterone system, immune modulation, attenuation of tubulointerstitial inflammation, and effects on podocytes. Published clinical trials have evaluated both native forms (cholecalciferol, ergocalciferol, and calcifediol) and active forms and analogs (calcitriol, paricalcitol, and alfacalcidol), with outcomes including urinary measures of albuminuria and proteinuria, renal function estimated by glomerular filtration rate and serum creatinine, metabolic control, inflammatory markers, and clinical events (6, 7).

Systematic reviews published in recent years consistently indicate an antiproteinuric signal of heterogeneous magnitude associated with vitamin D supplementation, particularly with active analogs such as paricalcitol and calcitriol, whereas effects on glomerular filtration and serum creatinine are less consistent (8–10). A recent tertiary synthesis reported a possible reduction in proteinuria without a demonstrated effect on other renal parameters (11). This dispersion of results reflects recognizable features of the primary evidence base: pharmacologic differences between native forms and active analogs, populations with different stages of kidney disease, inconsistent reporting of renoprotective cointerventions, and urinary outcomes defined non-equivalently across studies. The accumulation of systematic reviews with partially overlapping inclusion criteria over a short time span justifies a hierarchical reading of the aggregated body of secondary evidence, capable of distinguishing consistent findings from those supported by individual reviews and of contextualizing the nutrient effect against the current renoprotective standard: renin-angiotensin system blockade, sodium-glucose cotransporter 2 (SGLT2) inhibitors, selected glucagon-like peptide-1 receptor agonists (GLP-1 RAs), and nonsteroidal mineralocorticoid receptor antagonists.

The main objective of this umbrella review was to critically evaluate and summarize the efficacy and safety of vitamin D supplementation in adults with diabetic kidney disease (DKD), including studies reported as diabetic nephropathy (DN). The review aimed to distinguish evidence for native vitamin D from evidence for active vitamin D analogs where the secondary literature allowed separable interpretation, without assuming pharmacologic equivalence or formal comparative ranking in the absence of direct or network evidence. The research question was operationalized as follows: adult population with DKD/DN; intervention with native vitamin D or active forms/analogs; comparator of placebo, no intervention, standard care, active comparators, or different formulations and doses; and primary outcome of reduced albuminuria or proteinuria, assessed by urinary albumin-to-creatinine ratio (UACR), urinary albumin excretion rate (UAER), or 24-hour proteinuria. Secondary objectives were to synthesize effects on estimated glomerular filtration rate (eGFR), serum creatinine, kidney replacement therapy, mortality, cardiovascular events, blood pressure, lipid profile, glycemic control, inflammatory markers, quality of life, and safety; describe evidence separately for native forms versus active analogs when possible; and analyze sources of heterogeneity by formulation, kidney disease stage, baseline 25-hydroxyvitamin D [25(OH)D] status, dose, treatment duration, and cointerventions. The *a priori* hypothesis was conservative: vitamin D might reduce albuminuria or proteinuria as a surrogate outcome, but the available secondary evidence does not yet allow confirmation of clinical renoprotection on hard outcomes or conclusions regarding comparative safety.

## 2 Methods

### 2.1 Protocol registration and reporting guidelines

The protocol for this overview of reviews was registered in the International Prospective Register of Systematic Reviews (PROSPERO) under identifier CRD420251250914 on December 10, 2025. The report was structured according to the Preferred Reporting Items for Systematic Reviews and Meta-Analyses 2020 (PRISMA 2020) recommendations for general systematic review components (12) and the Preferred Reporting Items for Overviews of Reviews (PRIOR) recommendations for overviews of reviews (13). The completed PRIOR checklist is provided as **Supplementary Material 1**.

All operational stages were designed to be performed by two independent reviewers. Discrepancies in study selection, data extraction, methodological quality assessment with A MeaSurement Tool to Assess systematic Reviews 2 (AMSTAR-2) (14), risk of bias assessment with Risk Of Bias In Systematic reviews (ROBIS) (15), and certainty of evidence assessment with Grading of Recommendations Assessment, Development and Evaluation (GRADE) (16) were resolved by consensus until agreement was reached. This rule was applied uniformly to preserve traceability and reduce arbitrariness in the synthesis.

### 2.2 Research question and eligibility criteria

The question was operationalized using the Population-Intervention-(Exposure)-Comparator-Outcome [PI(E)CO] format. The population comprised adults aged 18 years or older with diabetic kidney disease (DKD), including studies reported as diabetic nephropathy (DN), defined by clinical diabetic etiology or histologic confirmation when available. Reviews with mixed populations were accepted only if DKD/DN-specific data were extractable, or if at least 80% of participants had DKD. The intervention included any form of vitamin D: native forms, including cholecalciferol, ergocalciferol, and calcifediol; and active forms or analogs, including calcitriol, paricalcitol, alfacalcidol, doxercalciferol, eldecalcitol, and maxacalcitol. Eligible comparators were placebo, usual care, no intervention, active controls, different doses, or comparisons between formulations. The primary outcome was reduction in albuminuria/proteinuria, measured as urinary albumin-to-creatinine ratio (UACR), urinary albumin excretion rate (UAER), or 24-hour proteinuria. Secondary outcomes included estimated glomerular filtration rate (eGFR), serum creatinine, creatinine clearance, kidney replacement therapy, all-cause and cardiovascular mortality, major adverse cardiovascular events, blood pressure, lipid profile, glycemic control, inflammatory or oxidative markers, quality of life, and safety.

Systematic reviews with or without quantitative meta-analysis that described a search strategy, eligibility criteria, and synthesis methods were included. Reviews that combined randomized controlled trials (RCTs) with nonrandomized studies were eligible only if the RCT component was separable for causal inference. Narrative reviews, scoping reviews, rapid reviews, commentaries, editorials, abstracts without full text, reviews based exclusively on observational studies, and reviews of multicomponent interventions in which the isolated effect of vitamin D could not be discerned were excluded. When duplicate reviews addressing the same question were available, the one closest to the PI(E)CO framework, with separable RCTs, compatible outcomes, more complete coverage, greater recency, and lower relative risk of bias was retained as the anchor review; the others were retained as confirmatory, contextual, or historical evidence.

### 2.3 Search strategy

The search strategy was designed to identify systematic reviews and meta-analyses on vitamin D in diabetic nephropathy or diabetic kidney disease. MEDLINE/PubMed, Embase, the Cochrane Library, Web of Science Core Collection, and Scopus were searched from inception to April 2, 2026. No language restrictions were applied. The strategy combined three conceptual blocks: diabetic nephropathy or diabetic kidney disease; vitamin D, including native forms and active analogs; and systematic review, meta-analysis, or overview study design. Medical Subject Headings (MeSH), Emtree terms (Embase), and free-text terms adapted to each database were used.

The supplementary search included manual screening of reference lists from the included reviews, citation tracking of key reviews, searches in PROSPERO, and other complementary sources when appropriate. Full search strategies for each database are provided in **Supplementary Material 2**.

### 2.4 Study selection and data extraction

Identified records were imported into Rayyan QCRI, with duplicates removed before screening. Two reviewers independently screened titles and abstracts; records deemed eligible or uncertain by at least one reviewer proceeded to full-text assessment. At the full-text stage, disagreements were resolved by consensus or arbitration by a third reviewer. Reasons for full-text exclusion were explicitly recorded and are reported in the **Supplementary Material**.

Data extraction was performed using a standardized form piloted in advance in Microsoft Excel. Minimum variables extracted from each review included first author, publication year, first author country when available, review type, databases searched, search date, protocol registration, number of primary studies, primary study designs, total number of participants, population criteria, vitamin D formulation, dose, route, duration, comparator, outcomes, pooled estimates with 95% confidence intervals (95% CIs), p value, statistical heterogeneity (I²), between-study variance (τ²) when available, meta-analytic model, variance estimation method, subgroup analyses, sensitivity analyses, publication bias assessment, renoprotective cointerventions, and adverse events. Extraction was performed in duplicate for the principal reviews, and discrepancies were resolved by consensus.

### 2.5 Assessment of methodological quality and risk of bias of the reviews

Methodological confidence in the included reviews was assessed with AMSTAR-2 (14). All 16 items were considered, with emphasis on the seven critical domains: prior protocol, comprehensive search, justification of excluded studies, risk of bias in primary studies, adequacy of meta-analytic methods, consideration of risk of bias when interpreting results, and assessment of publication bias. The global rating was applied strictly: high confidence if there were no critical flaws and at most one noncritical weakness; moderate if there was more than one noncritical weakness; low if there was one critical flaw with or without noncritical weaknesses; and critically low if there was more than one critical flaw. A more lenient operational classification could be retained only as a descriptive sensitivity analysis, not as the global AMSTAR-2 judgment.

ROBIS (15) was applied as a complementary tool for review-level risk of bias. The domains of eligibility, search and selection, extraction and appraisal, and synthesis of results were evaluated, with a global judgment for each review. Concordance between AMSTAR-2 and ROBIS was interpreted qualitatively, because AMSTAR-2 measures methodological confidence whereas ROBIS estimates the likelihood of bias in the review and its conclusions.

### 2.6 Assessment of overlap among primary studies

Overlap of primary studies across reviews was quantified using Corrected Covered Area (CCA) according to Pieper et al. (17). A citation matrix was constructed with rows representing unique primary studies and columns representing systematic reviews. CCA was calculated as (N−r)/(r×c−r), where N is the total number of occurrences of primary studies, r the number of unique primary studies, and c the number of reviews. The cutoffs used were <5% slight, 5–10% moderate, 11–15% high, and >15% very high.

The citation matrix included 49 unique primary studies and 115 occurrences across the 11 reviews considered for the overall corpus, yielding a CCA of 13.5%, consistent with high overlap. In the RCT-derived corpus, restricted to nine reviews with RCTs or separable randomized-trial data, 46 unique primary studies and 98 occurrences were identified, yielding a CCA of 14.1%, also consistent with high overlap. These values were treated as descriptive indicators of redundancy, not as a statistical test or an automatic exclusion criterion. The analytical consequence was to avoid pooling estimates from non-independent reviews and to prioritize an outcome-specific anchor-review strategy.

### 2.7 Data synthesis and quantitative reanalysis

The primary synthesis was structured by outcome and intervention type. No pharmacologic equivalence between native vitamin D and active analogs was assumed. UACR, UAER, 24-hour proteinuria, urinary protein-to-creatinine ratio (UPCR), and urinary protein concentrations were not indiscriminately pooled; they share a clinical direction, but they are not interchangeable metrics. The anchor review for each outcome was selected according to ordered operational criteria: proximity to the PI(E)CO framework, availability of RCTs or separable randomized-trial data, direct compatibility of the reported outcome, avoidance of double counting under high CCA, relative methodological quality and risk of bias, recency and completeness of coverage, and lower unexplained heterogeneity.

Anchor selection did not imply that non-anchor reviews were discarded; they were used to evaluate consistency, contextual applicability, and sensitivity of inference. Alternative anchor choices were considered qualitatively, but they did not alter the direction of the main conclusion because non-anchor estimates were either more heterogeneous, less outcome-specific, based on mixed designs, or dependent on *post hoc* sensitivity analyses.

This study did not include *de novo* quantitative reanalysis of primary studies. The synthesis is based on the estimates reported by the anchor reviews selected for each outcome, with critical assessment of overlap using CCA, statistical heterogeneity using I² and τ² when available, and certainty of evidence using GRADE. The decision not to conduct a new meta-analysis was based on two explicit considerations: the high overlap between reviews (overall CCA 13.5% and RCT-derived corpus CCA 14.1%) prevents treating the estimates as independent confirmations, and additional pooling of primary studies already extensively captured by the anchor reviews would introduce analytical redundancy without adding new causal inference. A full primary reanalysis by outcome was beyond the scope of this overview.

### 2.8 Classification of credibility and certainty of the evidence

Certainty of evidence was assessed with GRADE for each outcome (16). Evidence from RCTs started as high certainty and was downgraded for risk of bias, inconsistency, indirectness, imprecision, and publication bias. GRADE constitutes the definitive certainty assessment of this study.

## 3 Results

### 3.1 Search results and analytical corpus

**Figure 1:**
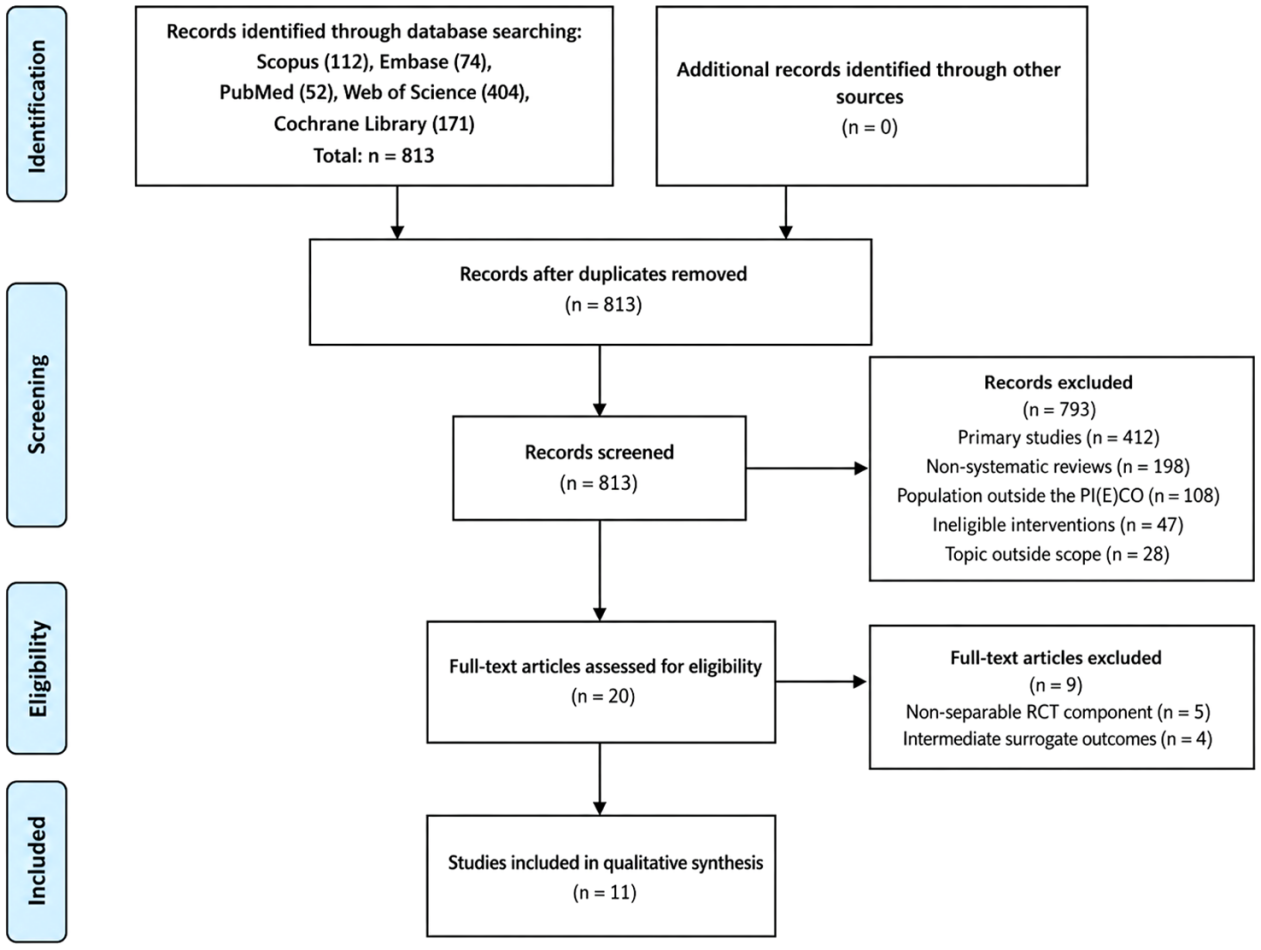
PRISMA 2020 flow diagram of the review selection process.

The selection process is summarized in **Figure 1**. A total of 813 records were identified through database searching: Scopus (n = 112), Embase (n = 74), PubMed (n = 52), Web of Science (n = 404), and the Cochrane Library (n = 171), with no additional records identified through other sources. No duplicate records were identified, so all 813 records proceeded to title and abstract screening. Of these, 793 were excluded at that stage. Thus, 20 full-text articles were assessed for eligibility, of which nine were excluded. Ultimately, 11 reviews met the inclusion criteria: nine contributed to the RCT-derived analytical corpus because they provided RCTs or separable randomized-trial data, and two were retained as contextual evidence due to mixed designs or incomplete separability (8–10, 18–25). **Table 1** presents the main characteristics of the included reviews and the analytical role assigned to each within the synthesis.

**Table 1:** Characteristics of the included reviews and analytical role.

Review	Type; studies/N	Population	Intervention	Analytical role	AMSTAR-2	ROBIS
Sharma 2023	SR RCTs; 5/377	T2DM + DKD	Native D3	Native narrative anchor	CB	AP
He 2022	SR/MA RCTs; 9/1547	DN/DKD	Native + analogs	UACR/UAER anchor	CB	High
Ullmann/Ramalho 2023	Mixed SR; 6/NR	DKD	Native + analogs	Contextual; separable RCTs	CB	High
Wang 2019	SR/MA RCTs; 20/1464	DN/DKD	Native + analogs	Secondary anchor	CB	High
Zhao 2014	SR/MA; 20/1497	DN/DKD	D3/analogs	Historical	CB	High
Gupta 2019	MA RCTs; 9/NR	DN/DKD	Vit D/analogs	Confirmatory	CB	High
Schuster 2019	Focused SR; 4/389	Residual albuminuria	Paricalcitol	RAAS context	CB	High
Derakhshanian 2015	Mixed SR/MA; 219 intervention	Diabetes + nephropathy	Vitamin D	Contextual	CB	High
Xuan 2023	SR/MA RCTs; 10/651	DN/DKD	Mixed vitamin D	Confirmatory	CB	High
Chokhandre 2015	Mixed SR; 6/NR	T2DM + DN	D3/calcitriol/paricalcitol	Contextual	CB	High
Uwaezuoke 2021	Mixed SR; 8/6243	DKD	Analogs	Pharmacologic context	CB	High

AMSTAR-2: CB = critically low. ROBIS: AP = some concerns. DKD, diabetic kidney disease; T2DM, type 2 diabetes mellitus; DN, diabetic nephropathy; RCTs, randomized controlled trials; MA, meta-analysis; NR, not reported; SR, systematic review. Detailed AMSTAR-2 and ROBIS matrices are presented in the **Supplementary Material**.

### 3.2 Synthesis of the primary outcome

The most consistent signal was observed in surrogate urinary outcomes. He 2022 was the anchor review for UACR and UAER because of outcome specificity, an RCT evidence base, and low-to-moderate heterogeneity. This signal supports reduced albuminuria, not proven clinical renoprotection. Estimates were reported as standardized mean differences (SMDs) when urinary metrics originated from heterogeneous scales, and as mean differences (MDs) when primary studies used a common metric. Wang 2019 provided favorable estimates for 24-hour proteinuria and UAER, but with extreme heterogeneity, and was therefore interpreted as a degraded secondary anchor. Xuan 2023 confirmed a potentially favorable direction, although its main results for proteinuria and creatinine depended on sensitivity analyses excluding an influential study. **Table 2** summarizes effect direction, the available estimate, heterogeneity, GRADE certainty, and the main reasons for downgrading each outcome.

**Table 2:** Synthesis of effects, certainty, and operational classification by outcome.

Outcome	Source	Estimate or summary	I²	Direction	GRADE	Clinical interpretation
UACR	He 2022	SMD -0.24 (95% CI -0.39 to -0.09); p=0.002	10%	Favorable	low	Antialbuminuric signal; not a hard outcome.
UAER	He 2022	SMD -0.57 (95% CI -0.71 to -0.43); p<0.00001	34%	Favorable	low	Antialbuminuric signal; short follow-up.
24-hour proteinuria	Wang 2019; Xuan 2023 sensitivity	MD -0.26 g/day (95% CI -0.34 to -0.17); Xuan favorable only after sensitivity analysis	95%; 93%->35%	Favorable/unstable	very low-low	Do not pool with UACR/UAER.
eGFR/creatinine	Wang/Xuan	No effect on eGFR; creatinine unstable and sensitive to influential studies	0%-variable	Uncertain	very low	Does not demonstrate renoprotection.
KRT, mortality, and MACE	No robust anchor	Data absent or insufficiently synthesized	NE	Not evaluable	very low	No clinical inference.
Safety	No robust anchor	Adverse events sparsely reported	NE	Insufficient	NE; very low	Absence of a signal does not demonstrate safety.
Metabolic/inflammatory	Complementary reviews	Heterogeneous and secondary results	NR	Uncertain	NS/NE; very low-low	Does not support a metabolic recommendation.

95% CI , 95% confidence interval; I², statistical inconsistency; MACE, major adverse cardiovascular events; MD, mean difference; NE, not evaluable; NR, not reported; SMD, standardized mean difference; KRT, kidney replacement therapy; UACR, urinary albumin-to-creatinine ratio; UAER, urinary albumin excretion rate. GRADE constitutes the definitive certainty assessment of this study. The clinical interpretation column reflects the inference that can be sustained given the available secondary evidence and the documented overlap among reviews.

### 3.3 Renal function, hard clinical outcomes, and safety

No robust evidence of preserved eGFR or stable reduction in serum creatinine was identified. Hard outcomes, including kidney replacement therapy, all-cause mortality, cardiovascular mortality, and major adverse cardiovascular events, were not reported or synthesized sufficiently for inference. Safety was treated as an independent outcome domain. Across the included review-level syntheses, adverse events were sparsely and heterogeneously reported, and no robust anchor review was available for comparative safety. Clinically relevant events, including hypercalcemia, hyperphosphatemia, changes in serum calcium/phosphate, PTH suppression, nephrolithiasis, treatment discontinuation, serious adverse events, and differential safety between native vitamin D and active analogs, were not consistently synthesized. Therefore, absence of a consistent harm signal should be interpreted as insufficient ascertainment rather than evidence of safety. **Table 3** summarizes the safety domains and the limits of review-level inference.

**Table 3:** Safety outcomes and interpretability by vitamin D formulation.

Safety domain	Native vitamin D	Active analogs	Review-level synthesis available?	Clinical interpretation
Hypercalcemia	Not reliably synthesized	Not reliably synthesized; clinically more relevant concern	No robust pooled estimate	Comparative risk cannot be inferred
Hyperphosphatemia	Not reliably synthesized	Not reliably synthesized; clinically relevant in CKD-MBD	No robust pooled estimate	Cannot exclude formulation-specific risk
Serum calcium/phosphate	Inconsistently reported	Inconsistently reported	Not harmonized across reviews	Monitoring implications cannot be quantified
PTH suppression	Not consistently synthesized	Relevant to calcitriol/paricalcitol physiology	No robust synthesis	Oversuppression risk not evaluable
Nephrolithiasis	Not systematically reported	Not systematically reported	No robust synthesis	Cannot infer stone risk
Treatment discontinuation	Incompletely reported	Incompletely reported	No robust synthesis	Tolerability comparison unavailable
Serious adverse events	Sparse reporting	Sparse reporting	No robust anchor	Absence of signal does not demonstrate safety
Native vs active analogs	Direct comparative safety unavailable	Direct comparative safety unavailable	No direct or network comparison	No formulation can be ranked as safer

CKD-MBD, chronic kidney disease-mineral and bone disorder; PTH, parathyroid hormone. The table summarizes availability and interpretability of safety domains at the review level; it is not a *de novo* adverse-event meta-analysis.

### 3.4 Overlap among primary studies

CCA showed substantial redundancy across reviews. The overall calculation was 13.5% and the calculation restricted to the RCT-derived corpus was 14.1%, both compatible with high overlap according to prespecified cutoffs. Therefore, the reviews should not be interpreted as independent confirmations of one another. **Table 4** presents the overall and RCT-derived corpus CCA calculations, together with their interpretation and use in the synthesis.

**Table 4:** Overlap of primary studies using corrected covered area.

Domain	No. reviews	Unique studies	Occurrences	CCA	Interpretation	Use in synthesis
Overall included reviews	11	49	115	13.5%	High	Includes the primary corpus and contextual reviews with identifiable primary studies.
RCT-derived corpus	9	46	98	14.1%	High	Restricted to reviews with RCTs or separable randomized-trial data.

CCA, Corrected Covered Area. Formula: CCA = (N-r)/(r×c-r), where N corresponds to the total number of occurrences of primary studies, r to the number of unique primary studies, and c to the number of reviews. The cutoffs used were <5% slight, 5–10% moderate, 11–15% high, and >15% very high.

### 3.5 Methodological quality and risk of bias of the reviews

When AMSTAR-2 was applied strictly, all reviews were classified as having critically low confidence, mainly because of the absence of a traceable prior protocol and the lack of a justified list of full-text excluded studies. ROBIS showed a predominance of high risk of bias, concentrated in the synthesis domain because of clinical and methodological heterogeneity, mixed study designs, and non-equivalent urinary metrics. Item- and domain-level details are presented outside the main text to avoid table overload: AMSTAR-2 in **Supplementary Material 6** and ROBIS in **Supplementary Material 7**.

### 3.6 Certainty of the evidence

**Figure 2:**
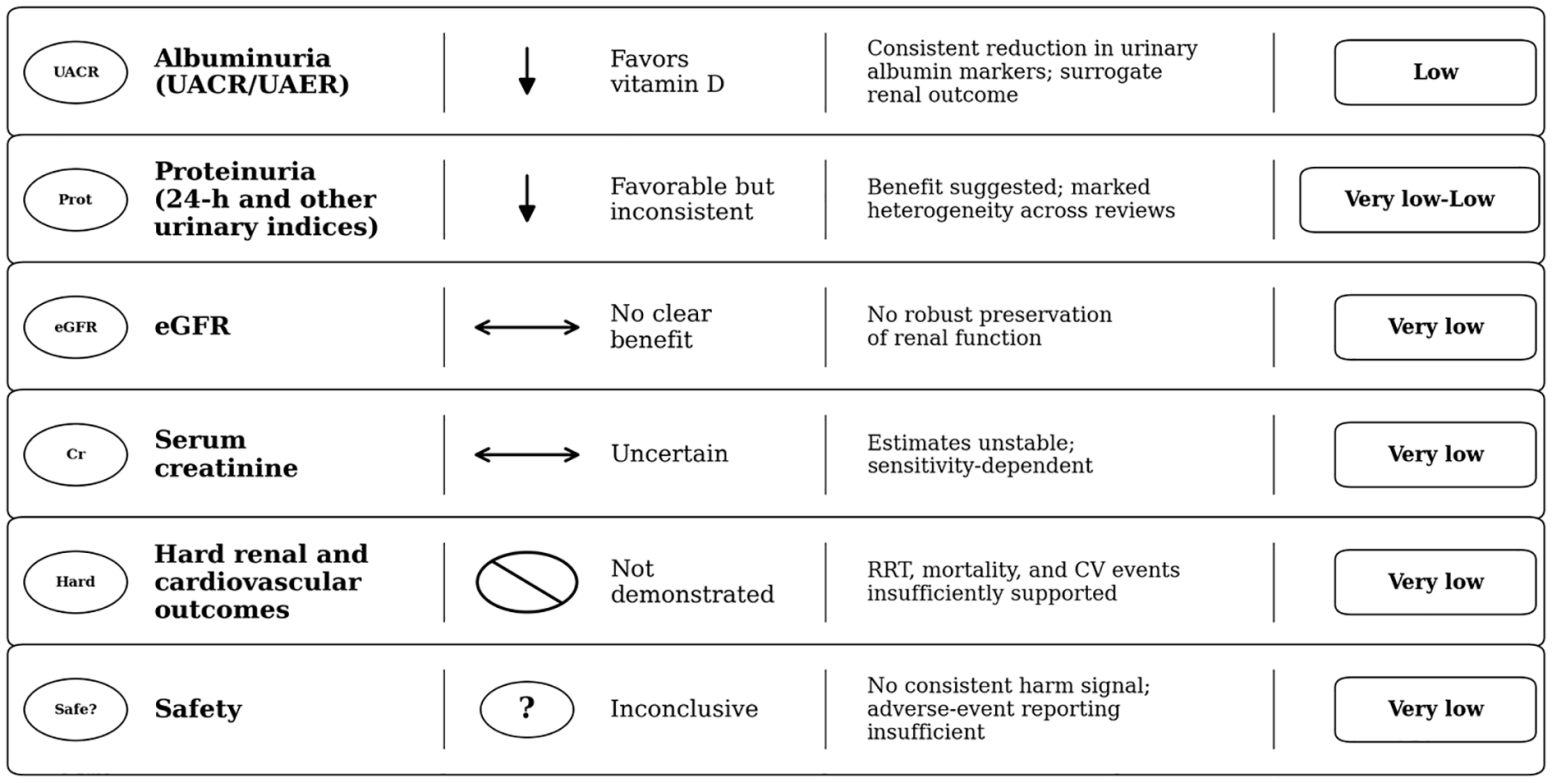
Certainty of evidence by outcome domain. Summary of effect direction and certainty of evidence by outcome domain. UACR, urinary albumin-to-creatinine ratio; UAER, urinary albumin excretion rate; eGFR, estimated glomerular filtration rate; KRT, kidney replacement therapy. Overall, the evidence suggests a low-certainty antiproteinuric signal without proven renoprotection for hard renal or cardiovascular outcomes.

GRADE was low for UACR and UAER, low to very low for 24-hour proteinuria, and very low or not evaluable for renal function, safety, and hard clinical outcomes. AMSTAR-2 and GRADE were interpreted as complementary rather than interchangeable judgments. Strict AMSTAR-2 ratings informed the risk-of-bias and interpretation domains, but they did not automatically determine the GRADE certainty for each outcome. UACR and UAER were retained as low rather than very low certainty because the anchor estimate was derived from randomized-trial evidence, was outcome-specific, showed low-to-moderate statistical heterogeneity, and had no major imprecision. Nevertheless, the evidence was downgraded for review-level methodological limitations, indirectness related to surrogate outcomes and heterogeneous DKD definitions, and possible publication bias. For 24-hour proteinuria, renal function, safety, and hard clinical outcomes, additional inconsistency, imprecision, sensitivity-dependent estimates, or absent synthesis justified very low or non-evaluable judgments. The complete GRADE profile by outcome is provided in **Supplementary Material 8**; key downgrading reasons are summarized in **Table 5**. The visual synthesis of effect direction and GRADE certainty by domain is presented in **Figure 2**, which distinguishes the favorable signal for albuminuria/proteinuria from the absence of robust evidence for renal function, hard outcomes, and safety.

**Table 5:** Main GRADE downgrading reasons by outcome domain.

Outcome/domain	GRADE certainty	Main downgrading reasons visible in the main manuscript
UACR	Low	Review-level risk of bias; surrogate-outcome indirectness; possible publication bias; no major imprecision; low heterogeneity.
UAER	Low	Review-level risk of bias; surrogate-outcome indirectness; possible publication bias; no major imprecision; low-to-moderate heterogeneity.
24-hour proteinuria	Very low-low	Risk of bias; extreme inconsistency; surrogate outcome; imprecision and sensitivity-dependent interpretation; possible publication bias.
eGFR/serum creatinine	Very low	Risk of bias; inconsistent or unstable estimates; imprecision; indirectness; possible publication bias.
KRT, mortality, MACE	Very low/not evaluable	Absent or insufficient synthesis; very serious imprecision; no robust anchor.
Safety	Very low/not evaluable	Sparse adverse-event reporting; no robust anchor; serious indirectness and imprecision; comparative safety not evaluable.
Metabolic/inflammatory outcomes	Very low-low	Secondary or heterogeneous outcomes; risk of bias; inconsistency; indirectness; imprecision; possible publication bias.

GRADE, Grading of Recommendations Assessment, Development and Evaluation; KRT, kidney replacement therapy; MACE, major adverse cardiovascular events; UACR, urinary albumin-to-creatinine ratio; UAER, urinary albumin excretion rate.

### 3.7 Clinical applicability and cointerventions

**Figure 3:**
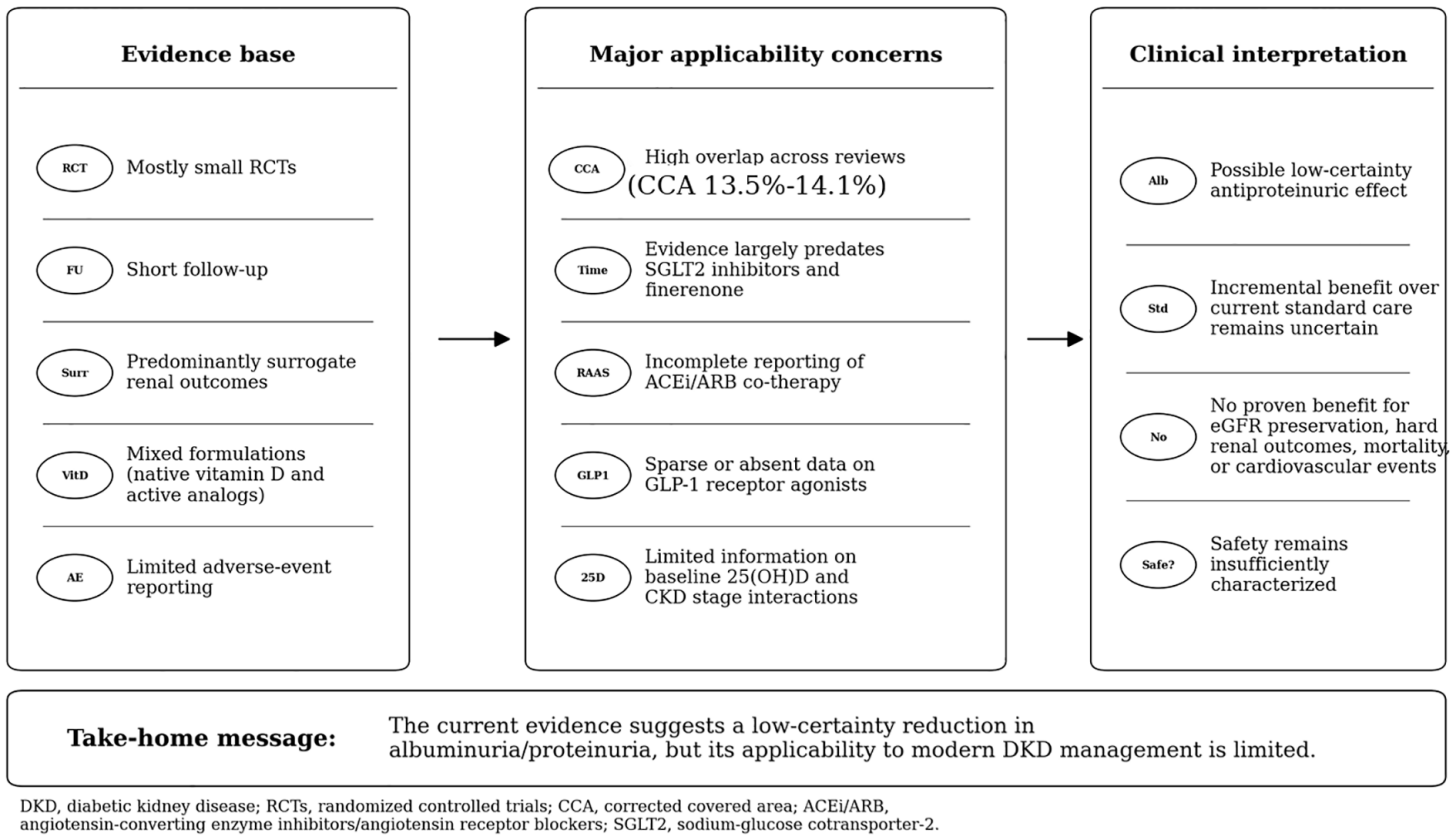
Applicability of the available evidence to contemporary management of diabetic kidney disease. Conceptual summary of the main applicability limitations of the available evidence. DKD, diabetic kidney disease; RCTs, randomized controlled trials; CCA, corrected covered area; ACEi/ARB, angiotensin-converting enzyme inhibitors/angiotensin receptor blockers; SGLT2, sodium-glucose cotransporter 2 inhibitors. Current evidence suggests a low-certainty reduction in albuminuria/proteinuria, but its incremental value over the contemporary standard of DKD care remains uncertain. CCA values in the figure were harmonized with the main text and tables as 13.5%-14.1%.

Applicability to the contemporary DKD standard of care is limited. Reporting of angiotensin-converting enzyme inhibitors (ACE inhibitors) and angiotensin II receptor blockers (ARBs) was incomplete, and the use of SGLT2 inhibitors, GLP-1 RAs, and nonsteroidal mineralocorticoid receptor antagonists was absent or not systematically reported. Consequently, the antiproteinuric signal should be interpreted as evidence generated in a predominantly pre-SGLT2 inhibitor and pre-finerenone context. Therefore, vitamin D should be interpreted primarily within conventional indications such as correction of vitamin D deficiency or management of CKD-mineral and bone disorder, not as an established adjunctive renoprotective therapy for contemporary DKD care. Whether vitamin D provides incremental renal benefit on top of current standard-of-care therapy remains an untested hypothesis rather than a clinical recommendation. **Figure 3** summarizes the clinical applicability problem: the evidence derives from small, short-term trials with predominantly surrogate outcomes and scant representation of the current therapeutic standard based on SGLT2 inhibitors, finerenone, and GLP-1 RAs.

Critical extraction corrections were preserved: Shab-Bidar 2011 corresponds to native vitamin D3 with calcium in fortified yogurt drink, not calcitriol; Ahmadi 2013 should be recorded with three months of follow-up; Barzegari/Esfandiari 2019 should be treated as 50,000 IU/week for eight weeks pending definitive primary verification; and VITAL-DKD/de Boer 2019 should be considered indirect evidence unless a DKD-specific subgroup is extracted.

### 3.8 Analytical sensitivities and synthesis of results

Analytical sensitivity checks supported the same conservative conclusion. First, restricting the synthesis to reviews with randomized trials or separable randomized-trial data did not materially change the interpretation of UACR or UAER, because He 2022 remained the outcome-specific anchor. Second, excluding indirect evidence without a DKD-specific subgroup did not alter the inference for renal function or hard outcomes, which remained absent or insufficiently synthesized. Third, assigning greater weight to Xuan 2023 would not strengthen the conclusion, because its findings for proteinuria and creatinine were sensitivity-dependent after removal of an influential study. Fourth, separating native vitamin D from active analogs prevented a class-effect claim: native vitamin D evidence was limited and mainly narrative, whereas active analog evidence suggested a possible antiproteinuric direction but did not establish comparative efficacy or safety. Fifth, high overlap was managed by avoiding pooled interpretation of non-independent review estimates and by selecting one anchor review per outcome; reviews were not treated as independent confirmations of one another.

Across these scenarios, the conclusion remained unchanged: vitamin D supplementation is associated with a low-certainty favorable signal in surrogate urinary outcomes, without demonstrated preservation of eGFR, reduction in kidney replacement therapy, mortality, cardiovascular events, or reliable comparative safety.

## 4 Discussion

### 4.1 Main findings

This overview evaluated seven outcome domains in vitamin D supplementation for diabetic kidney disease or diabetic nephropathy (DKD/DN). Only surrogate urinary outcomes reached low GRADE certainty with a favorable direction: UACR, with SMD −0.24 (95% CI −0.39 to −0.09); UAER, with SMD −0.57 (95% CI −0.71 to −0.43); and 24-hour proteinuria, with MD −0.26 g/day (95% CI −0.34 to −0.17), the latter with extreme heterogeneity and dependent on sensitivity analysis in one of the contributing reviews. The domains of renal function, kidney replacement therapy, mortality, major adverse cardiovascular events, safety, and metabolic-inflammatory outcomes were graded as very low certainty or classified as not evaluable because of insufficient synthesis. The central pattern, therefore, is not one of clinical renoprotective effect, but rather a low-certainty antiproteinuric and antialbuminuric signal concentrated in surrogate outcomes. The fact that no domain reached moderate or high certainty does not represent an analytical failure: it reflects the information that a rigorous overview should reveal when the secondary literature has been more abundant than conclusive.

### 4.2 Comparison with other studies and clinical implications

The findings are consistent with the most recent individual systematic reviews and with the overview of meta-analyses published in 2025: vitamin D and its analogs may reduce proteinuria and albuminuria, but they do not show a consistent effect on eGFR, creatinine, or hard clinical outcomes (8–11, 20). The main difference relative to those syntheses lies not in the direction of the urinary effect, but in the critical appraisal of the aggregated body of secondary evidence using strict AMSTAR-2, ROBIS, overlap quantification with CCA, and outcome-specific GRADE certainty. He 2022 provided the most specific estimate for UACR and UAER; Wang 2019 extended the domain toward 24-hour proteinuria and inflammatory markers, with very high heterogeneity; Xuan 2023 confirmed a favorable direction, although some of its results depended on sensitivity analyses after removal of an influential study (8, 10, 20). The Chackochan 2025 overview served to evaluate external consistency of effect direction, but it was not incorporated as an analytical unit because it constituted tertiary evidence; its inclusion would have introduced redundancy with the systematic reviews already included in the corpus.

Clinical heterogeneity also limits formulation-specific inference. Native vitamin D compounds and active vitamin D analogs differ in pharmacologic activation, calcium-phosphate effects, and clinical indications, and they should not be interpreted as a single interchangeable intervention. However, the included review-level evidence did not allow reliable estimation of treatment modification by CKD stage, baseline 25(OH)D status, diabetes type, albuminuria severity, dose, or treatment duration. Therefore, the present overview can describe the direction of evidence by outcome domain, but it cannot define the optimal compound, dose, duration, or target subgroup.

The biological plausibility of reduced albuminuria is reasonable. Vitamin D receptor activation modulates the renin-angiotensin-aldosterone system, tubulointerstitial inflammation, oxidative stress, and podocyte integrity--mechanisms consistent with reduced urinary albumin leakage (6, 7). Mechanistic plausibility, however, is not equivalent to clinical causality. Causal inference for hard renal outcomes requires temporal consistency, dose-response effects, adequate counterfactual frameworks, pragmatic trials with sufficient power for clinical outcomes, and, where applicable, triangulation with Mendelian randomization. In the available body of evidence, none of these requirements are met for outcomes beyond albuminuria and proteinuria; the very low GRADE certainty for renal function and clinical events directly reflects this limitation.

Similarly, reduction in albuminuria or proteinuria should not be interpreted as disease modification unless it is accompanied by evidence of slower eGFR decline, reduced kidney failure, fewer cardiovascular events, or lower mortality. In the present evidence base, the favorable direction is confined to urinary surrogate outcomes and does not establish clinical renal protection.

From a clinical standpoint, the current evidence does not justify recommending vitamin D as a specific renoprotective therapy to prevent eGFR decline, kidney replacement therapy, mortality, or cardiovascular events in DKD/DN. Vitamin D should be interpreted primarily within conventional indications such as correction of vitamin D deficiency or management of CKD-mineral and bone disorder, not as an established adjunctive renoprotective therapy for contemporary DKD care. The available urinary signal was generated in treatment contexts with incomplete ACE inhibitor/ARB reporting and little or no systematic representation of SGLT2 inhibitors, finerenone, or GLP-1 receptor agonists. Consequently, whether vitamin D provides incremental renal benefit on top of current standard-of-care therapy remains an untested hypothesis rather than a clinical recommendation (26, 27).

### 4.3 Study limitations

This study has several limitations. First, an overview inherits the quality of the included reviews; here, strict AMSTAR-2 classified all 11 reviews as critically low confidence, making any derived synthesis necessarily conservative. Second, the available corpus is based on small RCTs with short follow-up and predominantly surrogate outcomes; biologically real but clinically modest effects on renal function or hard outcomes may fail to reach statistical significance in low-powered aggregate meta-analyses, a limitation that GRADE captures through the imprecision domain but does not solve. Third, primary studies not included in systematic reviews are excluded by design from an overview. Fourth, small-study effects and excess significance tests, as reported by the source reviews, have low power when meta-analyses include few primary studies. Fifth, the search cutoff of April 2, 2026 means that later reviews were not incorporated. Sixth, the overall CCA of 13.5% and the RCT-derived corpus CCA of 14.1% indicate high overlap; therefore, conclusions should be interpreted as a hierarchical synthesis of redundant secondary evidence rather than repeated independent confirmation. Additional limitations include possible publication bias and selective reporting of favorable surrogate urinary outcomes in small vitamin D trials, limited follow-up for evaluating CKD progression, and the inability of the available review-level evidence to identify an optimal formulation, dose, treatment duration, or patient subgroup most likely to benefit.

## 5 Conclusions and recommendations

The clinically supportable conclusion is limited but useful: vitamin D or its analogs show a low-certainty antiproteinuric and antialbuminuric signal in adults with DKD, including studies reported as DN, but the evidence remains confined to surrogate urinary outcomes and does not establish preservation of renal function, reduction in hard renal events, mortality, cardiovascular events, or comparative safety. Future primary research should prioritize pragmatic, multicenter RCTs with adequate power, stratified by baseline 25(OH)D status, formulation (native versus active analogs), CKD stage, albuminuria category, and concomitant use of RAAS blockers, SGLT2 inhibitors, and finerenone, with sufficient follow-up to assess eGFR slope, renal progression, cardiovascular events, and calcium-phosphate safety. Until such evidence becomes available, vitamin D should not be promoted as a specific renoprotective intervention in DKD/DN; correction of a nutritional deficiency and protection of the kidney are distinct clinical hypotheses and should be treated as such.

## Data Availability

The original contributions presented in the study are included in the article/**Supplementary Material**. Further inquiries can be directed to the corresponding author.
