# Supplementary material for: Efficacy and safety of vitamin D supplementation in diabetic kidney disease: an umbrella review of systematic reviews and meta-analyses
Source: Front Nephrol. 2026 Jul 15;6:1883351. doi: 10.3389/fneph.2026.1883351 (PMC13414108; doi:10.3389/fneph.2026.1883351)
Supplement: Supplementary file 1 [file Table1.docx]

**Supplementary Material**


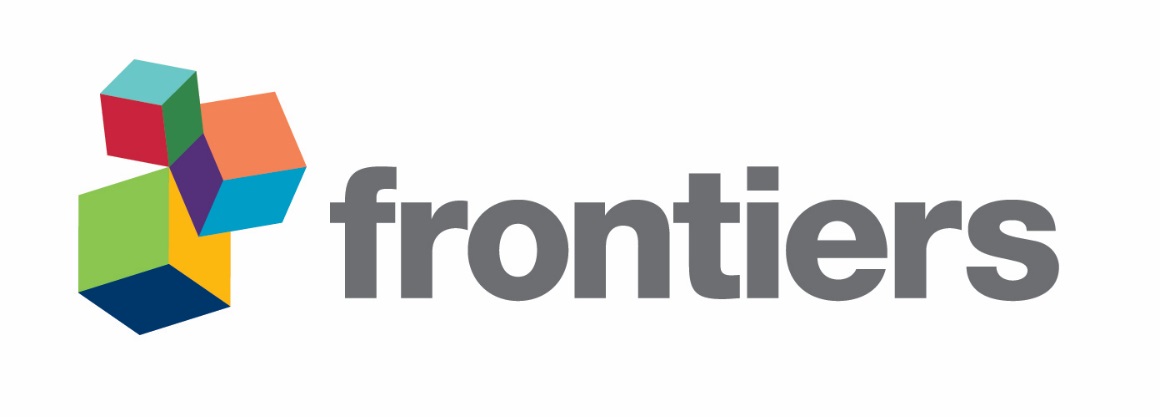


**Supplementary Material 1. PRIOR checklist**

**Supplementary Material 2. Search strategy by database**

**Supplementary Material 3. Evidence corpus layers and analytical restrictions**

**Supplementary Material 4. Complete characteristics of the included reviews**

**Supplementary Material 5. Overlap of primary studies using Corrected Covered Area**

**Supplementary Material 6. AMSTAR-2 by critical domains**

**Supplementary Material 7. ROBIS by domain and overall judgment**

**Supplementary Material 8. Complete GRADE profile by outcome**

**Supplementary Material 9. Analytical sensitivities and expected effect on the conclusion**

**Supplementary Material 10. Operational classification of credibility by outcome**

**Supplementary Material 11. Critical extraction and classification corrections**

**Supplementary Material 12. Cointerventions and applicability to the contemporary DKD standard**

**Supplementary Material 13. Traceability of anchor reviews by outcome**

**Supplementary Material**

**Supplementary Material 1. PRIOR checklist**

| **Section** | **Item** | **PRIOR item** | **Status** | **Location / note** |
| --- | --- | --- | --- | --- |
| TITLE | 1 | Identify the report as an overview of reviews, review of reviews, or umbrella review. | Yes | Manuscript title |
| ABSTRACT | 2 | Provide a comprehensive and accurate summary of the purpose, methods, and results of the overview of reviews. | Yes | Abstract |
| INTRODUCTION | 3 | Describe the rationale for the overview of reviews in the context of existing knowledge. | Yes | Introduction |
| INTRODUCTION | 4 | Provide an explicit statement of the objective or questions addressed by the overview of reviews. | Yes | Introduction, last paragraph |
| METHODS | 5a | Specify the inclusion and exclusion criteria for the overview of reviews. If supplementary primary studies were included, state and justify this. | Yes | Methods 2.2 |
| METHODS | 5b | Specify the definition of systematic review used in the inclusion criteria. | Yes | Methods 2.2 |
| METHODS | 6 | Specify all databases, registers, websites, organizations, reference lists, and other sources searched, as well as the date of the last search or consultation. | Yes | Methods 2.3; Supplementary Material 2 |
| METHODS | 7 | Present the full search strategies for all databases, registers, and websites in a reproducible manner, including any filters and limits applied. | Yes | Supplementary Material 2 |
| METHODS | 8a | Describe the methods used to decide whether a systematic review met the inclusion criteria. | Yes | Methods 2.4 |
| METHODS | 8b | Describe how overlap of populations, interventions, comparators, or outcomes across reviews was identified and managed during selection. | Yes | Methods 2.6; Supplementary Material 5 |
| METHODS | 9a | Describe the methods used to collect data from the reports. | Yes | Methods 2.4 |
| METHODS | 9b | Describe the methods used to identify and manage overlap of primary studies at the comparison and outcome level during data extraction. Specify the method used to illustrate or quantify overlap. | Yes | Methods 2.6; Results 3.4; Supplementary Material 5 |
| METHODS | 9c | Specify the methods used to manage discrepant data across systematic reviews during data extraction. | Yes | Methods 2.4 and 2.7 |
| METHODS | 10 | List and define all variables and outcomes for which data were sought. Describe assumptions or measures taken to clarify missing or unclear information. | Yes | Methods 2.4; registered protocol |
| METHODS | 11a | Describe the methods used to assess risk of bias or methodological quality of the included systematic reviews. | Yes | Methods 2.5; Supplementary Materials 6 and 7 |
| METHODS | 11b | Describe how risk-of-bias data for primary studies included in the systematic reviews were collected or assessed, and justify cases with incomplete or missing assessments. | Yes | Methods 2.5; Supplementary Materials 6-8 |
| METHODS | 11c | Describe the methods used to assess risk of bias in supplementary primary studies, if included. | NA | Supplementary primary studies were not included as an independent analytical unit |
| METHODS | 12a | Describe the methods used to summarize or synthesize the results and justify their choice. | Yes | Methods 2.7 |
| METHODS | 12b | Describe the methods used to explore possible causes of heterogeneity among results. | Yes | Methods 2.7; Supplementary Material 9 |
| METHODS | 12c | Describe the sensitivity analyses performed to assess the robustness of the synthesized results. | Yes | Methods 2.7; Supplementary Material 9 |
| METHODS | 13 | Describe the methods used to collect or assess risk of bias due to missing results in a synthesis, considering reviews, primary studies, and supplementary studies where applicable. | Yes | Methods 2.5 and 2.8; Supplementary Materials 6-8 |
| METHODS | 14 | Describe the methods used to collect or assess certainty or confidence in the body of evidence for each outcome. | Yes | Methods 2.8; Supplementary Material 8 |
| RESULTS | 15a | Describe the results of the search and selection process, including records screened, assessed for eligibility, and included, ideally with a flow diagram. | Yes | Results 3.1; Figure 1 |
| RESULTS | 15b | Provide a list of studies that may have appeared eligible but were excluded, together with the main reason for exclusion. | Yes | Results 3.1; reasons for exclusion in Figure 1 |
| RESULTS | 16 | Cite each included systematic review and supplementary primary study, if applicable, and present its characteristics. | Yes | Table 1; Supplementary Material 4 |
| RESULTS | 17 | Describe the degree of overlap of primary studies across the included systematic reviews. | Yes | Results 3.4; Table 3; Supplementary Material 5 |
| RESULTS | 18a | Present the risk-of-bias or methodological-quality assessments for each included systematic review. | Yes | Results 3.5; Supplementary Materials 6 and 7 |
| RESULTS | 18b | Present the risk-of-bias assessments for primary studies included in the systematic reviews, whether extracted from the reviews or reassessed. | Yes | Supplementary Materials 6-8 |
| RESULTS | 18c | Present the risk-of-bias assessments for supplementary primary studies, if included. | NA | Supplementary primary studies were not included |
| RESULTS | 19a | For all outcomes, summarize the evidence from systematic reviews and supplementary primary studies, if applicable. If meta-analyses were conducted, present the pooled estimate, precision, heterogeneity, and direction of effect. | Yes | Results 3.2-3.8; Tables 2-5; Figure 2 |
| RESULTS | 19b | If meta-analyses were conducted, present the results of all investigations into possible causes of heterogeneity. | NA | No de novo meta-analysis was performed in the main text |
| RESULTS | 19c | If meta-analyses were conducted, present the results of all sensitivity analyses performed to assess the robustness of the synthesized results. | Yes | Results 3.8; Supplementary Material 9 |
| RESULTS | 20 | Present assessments of risk of bias due to missing studies, analyses, or results in each assessed synthesis. | Yes | Results 3.5-3.6; Supplementary Materials 6-8 |
| RESULTS | 21 | Present the certainty or confidence assessments for the body of evidence for each outcome. | Yes | Results 3.6; Figure 2; Supplementary Material 8 |
| DISCUSSION | 22a | Summarize the main findings, including discrepancies between systematic reviews and supplementary primary studies, if applicable. | Yes | Discussion, first and second paragraphs |
| DISCUSSION | 22b | Provide an overall interpretation of the results in the context of other evidence. | Yes | Discussion |
| DISCUSSION | 22c | Discuss the limitations of the evidence from the reviews, their primary studies, and supplementary studies, as well as methodological limitations of the overview. | Yes | Discussion, limitations |
| DISCUSSION | 22d | Discuss implications for practice, policy, and future research, considering relevance to end users. | Yes | Discussion, clinical implications and conclusion |
| OTHER INFORMATION | 23a | Provide registration information for the overview, including registry name and number, or state that it was not registered. | Yes | Methods 2.1; PROSPERO CRD420251250914 |
| OTHER INFORMATION | 23b | Indicate where the overview protocol can be accessed or state that no protocol was prepared. | Yes | Methods 2.1; PROSPERO |
| OTHER INFORMATION | 23c | Describe and explain amendments to the registered information or protocol, indicating when they were made. | Yes | Methods 2.1; no substantive amendments reported |
| OTHER INFORMATION | 24 | Describe sources of financial or nonfinancial support for the overview and the role of funders or sponsors. | Yes | Funding information reported in manuscript; funder had no role in review conduct. |
| OTHER INFORMATION | 25 | Declare conflicts of interest of the overview authors. | Yes | Conflict-of-interest statement reported in manuscript; no conflicts declared. |
| OTHER INFORMATION | 26a | Provide contact information for the corresponding author. | Yes | Cover page/submission editorial information |
| OTHER INFORMATION | 26b | Describe individual author contributions and identify the guarantor of the overview. | Yes | Submission editorial information |
| OTHER INFORMATION | 27 | Report which data, code, and other materials are available, where they can be found, and under what conditions they can be accessed. | Yes | Supplementary Materials 5 and 8; matrices available upon reasonable request/repository |

**Supplementary Material 2. Search strategy by database**

| **Database** | **Search strategy** |
| --- | --- |
| MEDLINE/PubMed | ("Diabetic Nephropathies"[Mesh] OR "diabetic nephropathy"[tiab] OR "diabetic kidney disease"[tiab] OR "diabetic renal disease"[tiab] OR "diabetic glomerulosclerosis"[tiab] OR "Kimmelstiel-Wilson"[tiab] OR ((diabetes[tiab] OR diabetic[tiab]) AND (nephropathy[tiab] OR "kidney disease"[tiab] OR "renal disease"[tiab] OR albuminuria[tiab] OR proteinuria[tiab]))) AND ("Vitamin D"[Mesh] OR "Cholecalciferol"[Mesh] OR "Ergocalciferols"[Mesh] OR "Calcifediol"[Mesh] OR "Calcitriol"[Mesh] OR "vitamin D"[tiab] OR "vitamin D2"[tiab] OR "vitamin D3"[tiab] OR cholecalciferol[tiab] OR ergocalciferol[tiab] OR calcifediol[tiab] OR calcidiol[tiab] OR "25-hydroxyvitamin D"[tiab] OR "25(OH)D"[tiab] OR calcitriol[tiab] OR "1,25-dihydroxyvitamin D"[tiab] OR paricalcitol[tiab] OR alfacalcidol[tiab] OR doxercalciferol[tiab] OR eldecalcitol[tiab] OR maxacalcitol[tiab] OR "vitamin D receptor activator"[tiab] OR "VDRA"[tiab]) AND ("Meta-Analysis"[Publication Type] OR "Systematic Review"[Publication Type] OR "Meta-Analysis as Topic"[Mesh] OR "systematic review"[tiab] OR "meta-analysis"[tiab] OR metaanalysis[tiab] OR "umbrella review"[tiab] OR overview[tiab]) |
| Embase | #1 'diabetic nephropathy'/exp; #2 (diabetic NEAR/3 (nephropathy OR 'kidney disease' OR 'renal disease' OR glomerulosclerosis)):ti,ab,kw; #3 'kimmelstiel-wilson':ti,ab,kw; #4 ((diabetes OR diabetic) NEAR/3 (nephropathy OR kidney OR renal OR albuminuria OR proteinuria)):ti,ab,kw; #5 #1 OR #2 OR #3 OR #4; #6 'vitamin d'/exp; #7 'cholecalciferol'/exp; #8 'ergocalciferol'/exp; #9 'calcifediol'/exp; #10 'calcitriol'/exp; #11 'paricalcitol'/exp; #12 'alfacalcidol'/exp; #13 'doxercalciferol'/exp; #14 ('vitamin d' OR 'vitamin d2' OR 'vitamin d3'):ti,ab,kw; #15 (cholecalciferol OR ergocalciferol OR calcifediol OR calcidiol):ti,ab,kw; #16 ('25-hydroxyvitamin d' OR '25(oh)d'):ti,ab,kw; #17 (calcitriol OR '1,25-dihydroxyvitamin d'):ti,ab,kw; #18 (paricalcitol OR alfacalcidol OR doxercalciferol OR eldecalcitol OR maxacalcitol):ti,ab,kw; #19 ('vitamin d receptor activator' OR vdra):ti,ab,kw; #20 #6 OR #7 OR #8 OR #9 OR #10 OR #11 OR #12 OR #13 OR #14 OR #15 OR #16 OR #17 OR #18 OR #19; #21 'systematic review'/exp; #22 'meta analysis'/exp; #23 ('systematic review' OR 'meta-analysis' OR metaanalysis OR 'umbrella review'):ti,ab,kw; #24 #21 OR #22 OR #23; #25 #5 AND #20 AND #24 |
| Scopus | TITLE-ABS-KEY("diabetic nephropathy" OR "diabetic kidney disease" OR "diabetic renal disease" OR "diabetic glomerulosclerosis" OR "Kimmelstiel-Wilson" OR ((diabetes OR diabetic) W/3 (nephropathy OR "kidney disease" OR "renal disease" OR albuminuria OR proteinuria))) AND TITLE-ABS-KEY("vitamin D" OR "vitamin D2" OR "vitamin D3" OR cholecalciferol OR ergocalciferol OR calcifediol OR calcidiol OR "25-hydroxyvitamin D" OR "25(OH)D" OR calcitriol OR "1,25-dihydroxyvitamin D" OR paricalcitol OR alfacalcidol OR doxercalciferol OR eldecalcitol OR maxacalcitol OR "vitamin D receptor activator" OR VDRA) AND TITLE-ABS-KEY("systematic review" OR "meta-analysis" OR metaanalysis OR "umbrella review" OR overview) |
| Web of Science Core Collection | TS=("diabetic nephropathy" OR "diabetic kidney disease" OR "diabetic renal disease" OR "diabetic glomerulosclerosis" OR "Kimmelstiel-Wilson" OR ((diabetes OR diabetic) NEAR/3 (nephropathy OR "kidney disease" OR "renal disease" OR albuminuria OR proteinuria))) AND TS=("vitamin D" OR "vitamin D2" OR "vitamin D3" OR cholecalciferol OR ergocalciferol OR calcifediol OR calcidiol OR "25-hydroxyvitamin D" OR "25(OH)D" OR calcitriol OR "1,25-dihydroxyvitamin D" OR paricalcitol OR alfacalcidol OR doxercalciferol OR eldecalcitol OR maxacalcitol OR "vitamin D receptor activator" OR VDRA) AND TS=("systematic review" OR "meta-analysis" OR metaanalysis OR "umbrella review" OR overview) |
| Cochrane Library | #1 [mh "Diabetic Nephropathies"]; #2 (diabetic NEAR/3 (nephropathy OR "kidney disease" OR "renal disease" OR glomerulosclerosis)):ti,ab,kw; #3 "Kimmelstiel-Wilson":ti,ab,kw; #4 ((diabetes OR diabetic) NEAR/3 (nephropathy OR kidney OR renal OR albuminuria OR proteinuria)):ti,ab,kw; #5 #1 OR #2 OR #3 OR #4; #6 [mh "Vitamin D"]; #7 [mh "Cholecalciferol"]; #8 [mh "Ergocalciferols"]; #9 [mh "Calcifediol"]; #10 [mh "Calcitriol"]; #11 ("vitamin D" OR "vitamin D2" OR "vitamin D3"):ti,ab,kw; #12 (cholecalciferol OR ergocalciferol OR calcifediol OR calcidiol):ti,ab,kw; #13 ("25-hydroxyvitamin D" OR "25(OH)D"):ti,ab,kw; #14 (calcitriol OR "1,25-dihydroxyvitamin D"):ti,ab,kw; #15 (paricalcitol OR alfacalcidol OR doxercalciferol OR eldecalcitol OR maxacalcitol):ti,ab,kw; #16 ("vitamin D receptor activator" OR VDRA):ti,ab,kw; #17 #6 OR #7 OR #8 OR #9 OR #10 OR #11 OR #12 OR #13 OR #14 OR #15 OR #16; #18 #5 AND #17 |

Note. Strategies derived from the protocol and the search file. The search was conducted from database inception to April 2, 2026, without language restrictions.

**Supplementary Material 3. Evidence corpus layers and analytical restrictions**

| **Layer** | **Reviews** | **Analytical use** | **Justification** |
| --- | --- | --- | --- |
| RCT-derived corpus | Sharma 2023; He 2022; Wang 2019; Zhao 2014; Gupta 2019; Schuster 2019; Xuan 2023; mixed reviews only if RCTs are separable | Primary outcome-specific synthesis | Provides RCTs or separable RCT data for efficacy inference. |
| Contextual evidence | Chokhandre 2015; Uwaezuoke 2021; non-separable components of Ullmann/Ramalho 2023 or Derakhshanian 2015 | Pharmacologic context, applicability, and discussion | Mixed designs or incomplete separability; should not govern causal inference. |
| Excluded tertiary evidence | Chackochan 2025 | External discussion, not primary synthesis | Overview of meta-analyses; would duplicate the unit of analysis of an umbrella review. |

Note. RCT = randomized controlled trial. Classification must remain traceable to full-text review in the final version.

**Supplementary Material 4. Complete characteristics of the included reviews**

| **Review** | **Type; studies/N** | **Population** | **Intervention** | **Analytical role** | **AMSTAR-2** | **ROBIS** |
| --- | --- | --- | --- | --- | --- | --- |
| Sharma 2023 | SR RCTs; 5/377 | T2DM + DKD | Native D3 | Native narrative anchor | CB | AP |
| He 2022 | SR/MA RCTs; 9/1547 | DN/DKD | Native + analogs | UACR/UAER anchor | CB | High |
| Ullmann/Ramalho 2023 | Mixed SR; 6/NR | DKD | Native + analogs | Contextual; separable RCTs | CB | High |
| Wang 2019 | SR/MA RCTs; 20/1464 | DN/DKD | Native + analogs | Secondary anchor | CB | High |
| Zhao 2014 | SR/MA; 20/1497 | DN/DKD | D3/analogs | Historical | CB | High |
| Gupta 2019 | MA RCTs; 9/NR | DN/DKD | Vit D/analogs | Confirmatory | CB | High |
| Schuster 2019 | Focused SR; 4/389 | Residual albuminuria | Paricalcitol | RAAS context | CB | High |
| Derakhshanian 2015 | Mixed SR/MA; 219 intervention | Diabetes + nephropathy | Vitamin D | Contextual | CB | High |
| Xuan 2023 | SR/MA RCTs; 10/651 | DN/DKD | Mixed vitamin D | Confirmatory | CB | High |
| Chokhandre 2015 | Mixed SR; 6/NR | T2DM + DN | D3/calcitriol/paricalcitol | Contextual | CB | High |
| Uwaezuoke 2021 | Mixed SR; 8/6243 | DKD | Analogs | Pharmacologic context | CB | High |

Note. Expanded table removed from the main text to reduce table burden. CB = critically low; AP = some concerns; NR = not reported.

**Supplementary Material 5. Overlap of primary studies using Corrected Covered Area**

| **Domain** | **No. reviews** | **Unique studies** | **Occurrences** | **CCA** | **Interpretation** | **Use in synthesis** |
| --- | --- | --- | --- | --- | --- | --- |
| Overall included reviews | 11 | 49 | 115 | 13.5% | High | Includes the primary corpus and contextual reviews with identifiable primary studies. |
| RCT-derived corpus | 9 | 46 | 98 | 14.1% | High | Restricted to reviews with RCTs or separable RCT data. |

Note. CCA = Corrected Covered Area. CCA below 5% = slight; 5–10% = moderate; 11–15% = high; >15% = very high. The citation matrix was constructed with rows for unique primary studies and columns for the included reviews.

**Supplementary Material 6. AMSTAR-2 by critical domains**

| **Review** | **Protocol** | **Search** | **Excluded** | **Primary RoB** | **MA method** | **RoB interp.** | **Pub. bias** | **Overall** |
| --- | --- | --- | --- | --- | --- | --- | --- | --- |
| Sharma 2023 | N | Y | N | PY | NA | PY | NA | CB |
| He 2022 | N | Y | N | Y | PY | PY | PY | CB |
| Ullmann/Ramalho 2023 | N | PY | N | PY | NA | PY | NA | CB |
| Wang 2019 | N | Y | N | Y | PY | PY | PY | CB |
| Zhao 2014 | N | PY | N | PY | PY | PY | N | CB |
| Gupta 2019 | N | PY | N | PY | NA | PY | NA | CB |
| Schuster 2019 | N | PY | N | PY | NA | PY | NA | CB |
| Derakhshanian 2015 | N | PY | N | PY | NA | PY | NA | CB |
| Xuan 2023 | N | Y | N | Y | PY | PY | PY | CB |
| Chokhandre 2015 | N | PY | N | PY | NA | PY | NA | CB |
| Uwaezuoke 2021 | N | PY | N | PY | NA | PY | NA | CB |

Note. Y = yes; PY = partially yes; N = no; NA = not applicable; CB = critically low; MA = meta-analysis; RoB = risk of bias. The overall rating uses strict AMSTAR-2.

**Supplementary Material 7. ROBIS by domain and overall judgment**

| **Review** | **D1 eligibility** | **D2 search/selection** | **D3 extraction/appraisal** | **D4 synthesis** | **Overall** |
| --- | --- | --- | --- | --- | --- |
| Sharma 2023 | Low | Low | AP | AP | AP |
| He 2022 | Low | Low | AP | High | High |
| Ullmann/Ramalho 2023 | AP | AP | AP | High | High |
| Wang 2019 | Low | AP | AP | High | High |
| Zhao 2014 | AP | AP | High | High | High |
| Gupta 2019 | AP | AP | AP | High | High |
| Schuster 2019 | AP | AP | AP | High | High |
| Derakhshanian 2015 | High | AP | High | High | High |
| Xuan 2023 | Low | AP | AP | High | High |
| Chokhandre 2015 | AP | AP | High | High | High |
| Uwaezuoke 2021 | High | AP | High | High | High |

Note. AP = some concerns. ROBIS evaluates review-level risk of bias and does not substitute for AMSTAR-2.

**Supplementary Material 8. Complete GRADE profile by outcome**

| **Outcome** | **Basis/anchor** | **Direction** | **RoB** | **Inconsistency** | **Indirectness** | **Imprecision** | **Pub. bias** | **Certainty** |
| --- | --- | --- | --- | --- | --- | --- | --- | --- |
| Albuminuria/proteinuria | He/Wang/Xuan | Nonuniform favorable | Serious | Serious | Serious | Variable | Possible | Low |
| UACR/UAER | He 2022 | Favorable | Serious | Not serious-serious | Serious | Not serious | Possible | Low |
| 24-hour proteinuria | Wang/Xuan | Favorable/unstable | Serious | Serious | Serious | Serious | Possible | Very low-low |
| eGFR/creatinine | Wang/Xuan | Null or uncertain | Serious | Serious | Serious | Serious | Possible | Very low |
| KRT/mortality/MACE | No anchor | Not evaluable | Very serious | NE | Serious | Very serious | NE | Very low |
| Safety | Sparse reporting | Insufficient | Serious | Serious | Serious | Serious | NE | Very low |
| Metabolic/inflammatory | Secondary | Inconsistent | Serious | Serious | Serious | Serious | Possible | Very low-low |

Note. GRADE was applied per outcome. MACE = major adverse cardiovascular events; NE = not evaluable; RoB = risk of bias; KRT = kidney replacement therapy.

**Supplementary Material 9. Analytical sensitivities and expected effect on the conclusion**

| **Sensitivity** | **Rationale** | **Expected effect on the conclusion** |
| --- | --- | --- |
| Exclude mixed reviews | Avoids causal inference based on nonseparable nonrandomized designs. | The UACR/UAER signal persists as low certainty; safety and hard outcomes remain unproven. |
| Exclude indirect VITAL-DKD-type evidence without a DKD subgroup | Avoids extrapolation from general type 2 diabetes to established DKD. | The conclusion does not change: eGFR and hard renal outcomes remain uncertain. |
| Separate native vitamin D vs active analogs | Cholecalciferol, calcitriol, and paricalcitol are not pharmacologically equivalent. | Prevents claiming superiority of one class without robust direct comparison. |
| Use strict AMSTAR-2 | The original rule classifies any review with more than one critical flaw as critically low. | All reviews remain critically low; the conclusion remains conservative. |
| Exclude results dependent on post hoc sensitivity analyses | Prevents an unstable analysis from dominating the conclusion. | Xuan 2023 remains confirmatory, not an anchor. |
| Outcome-specific anchor review | Avoids redundant pooling of non-independent estimates. | The synthesis focuses on direction, consistency, CCA, and GRADE. |

Note. The sensitivities distinguish RCT-derived inference, contextual evidence, and population indirectness.

**Supplementary Material 10. Operational classification of credibility by outcome**

| **Outcome or estimate** | **Main source** | **Summary result** | **GRADE** | **Interpretation** |
| --- | --- | --- | --- | --- |
| UACR | He 2022 | SMD -0.24 (95% CI -0.39 to -0.09); p=0.002; I2=10% | Low | Antialbuminuric signal; surrogate outcome. |
| UAER | He 2022 | SMD -0.57 (95% CI -0.71 to -0.43); p<0.00001; I2=34% | Low | More consistent signal; do not extrapolate to hard outcomes. |
| 24-hour proteinuria | Wang 2019 | MD -0.26 g/day (95% CI -0.34 to -0.17); I2=95% | Low | Favorable but highly heterogeneous. |
| Total proteinuria | Xuan 2023 | Initial result not significant; favorable after excluding an influential study | Very low-low | Confirmatory; not the primary anchor. |
| Serum creatinine | Wang/Xuan | Null or sensitivity-dependent | Very low | Does not demonstrate renoprotection. |
| eGFR | Wang 2019 | MD +2.13 (95% CI -2.06 to 6.32); p=0.32 | Very low | No robust preservation of renal function. |
| Glycemic control | Wang and others | No consistent significant differences | Very low | Does not support a metabolic recommendation. |
| Safety | No robust anchor | Adverse events sparsely reported | Very low | Absence of a signal does not demonstrate safety. |
| KRT, mortality, and MACE | No robust anchor | Data absent or not sufficiently synthesized | Very low | Not evaluable for hard clinical outcomes. |

Note. The classification is operational and should be updated when the primary-data reanalysis is finalized. NE = not evaluable; NS = not significant.

**Supplementary Material 11. Critical extraction and classification corrections**

| **Trial/record** | **Applied correction** | **Analytical impact** |
| --- | --- | --- |
| Shab-Bidar 2011 | Classify as native vitamin D3 with calcium in fortified yogurt drink, not as calcitriol. | Prevents contamination of the active-analog stratum. |
| Ahmadi 2013 | Record three months of follow-up. | Corrects inconsistency in follow-up duration. |
| Barzegari/Esfandiari 2019 | Treat as 50,000 IU/week for eight weeks pending definitive primary verification. | Prevents a 10-fold dosing error. |
| Krairittichai 2012 | Record calcitriol in micrograms, not milligrams. | Prevents a pharmacologically implausible unit. |
| VITAL-DKD/de Boer 2019 | Consider as indirect evidence unless a DKD-specific subgroup is available. | Prevents extrapolation from general type 2 diabetes to established DKD. |

Note. These corrections protect pharmacologic stratification and the external validity of the inference.

**Supplementary Material 12. Cointerventions and applicability to the contemporary DKD standard**

| **Cointervention** | **Reporting in the reviews** | **Implication for external validity** |
| --- | --- | --- |
| ACEi/ARB | Incomplete or heterogeneous. | Makes it difficult to determine whether the urinary effect is incremental over RAAS blockade. |
| SGLT2i | Absent or not systematically reported. | The evidence largely arises from a pre-SGLT2i context. |
| GLP-1 agonists | Absent or not systematically reported. | Does not allow assessment of interaction with contemporary cardiometabolic strategies. |
| Nonsteroidal mineralocorticoid antagonists/finerenone | Absent or not systematically reported. | The incremental value of vitamin D over the current standard remains undetermined. |
| Calcium and concomitant supplements | Variable; relevant in fortified interventions. | May modify mineral-bone safety and interpretation of causality. |

Note. DKD = diabetic kidney disease; GLP-1 = glucagon-like peptide-1; ACEi = angiotensin-converting enzyme inhibitor; RAAS = renin–angiotensin–aldosterone system; SGLT2i = sodium-glucose cotransporter-2 inhibitor.

**Supplementary Material 13. Traceability of anchor reviews by outcome**

| **Outcome/domain** | **Anchor review** | **Reason for selection** | **Restriction** |
| --- | --- | --- | --- |
| UACR/UAER | He 2022 | Greatest specificity for urinary albuminuria, an RCT evidence base, and low-to-moderate heterogeneity. | Do not extrapolate to hard renal outcomes. |
| Native vitamin D | Sharma 2023 | Recent source focused on native vitamin D. | Mainly narrative synthesis; do not use as a single quantitative estimator. |
| 24-hour proteinuria/renal function/inflammation | Wang 2019 | Broad meta-analysis for domains not covered by He 2022. | Secondary anchor downgraded because of extreme heterogeneity in some outcomes. |
| Confirmatory results | Xuan 2023 | Recent eligible review with RCTs. | Does not displace the anchor when effects depend on sensitivity analyses. |
| Safety | No robust anchor | Insufficient and heterogeneous adverse event reporting. | Do not conclude comparative safety. |

Note. The anchor review was selected based on PICO proximity, separability of RCTs, outcome compatibility, heterogeneity, recency, and relative risk of bias.
